# Gene Expression of Diverse Cryptococcus Isolates during Infection of the Human Central Nervous System

**DOI:** 10.1128/mBio.02313-21

**Published:** 2021-11-02

**Authors:** Chen-Hsin Yu, Poppy Sephton-Clark, Jennifer L. Tenor, Dena L. Toffaletti, Charles Giamberardino, Miriam Haverkamp, Christina A. Cuomo, John R. Perfect

**Affiliations:** a Division of Infectious Diseases, Department of Medicine, Duke University School of Medicine, Durham, North Carolina, USA; b Institute of Molecular Biology, Academia Sinica, Taipei, Taiwan; c Infectious Disease and Microbiome Program, Broad Institute of MIT and Harvard, Cambridge, Massachusetts, USA; d Department of Infection Control and Infectious Diseases, University Hospital RWTH Aachen, Aachen, Germany; Yonsei University

**Keywords:** *Cryptococcus neoformans*, meningitis, genes, transcription, human disease

## Abstract

Cryptococcus neoformans is a major human central nervous system (CNS) fungal pathogen causing considerable morbidity and mortality. In this study, we provide the widest view to date of the yeast transcriptome directly from the human subarachnoid space and within cerebrospinal fluid (CSF). We captured yeast transcriptomes from C. neoformans of various genotypes in 31 patients with cryptococcal meningoencephalitis as well as several Cryptococcus gattii infections. Using transcriptome sequencing (RNA-seq) analyses, we compared the *in vivo* yeast transcriptomes to those from other environmental conditions, including *in vitro* growth on nutritious media or artificial CSF as well as samples collected from rabbit CSF at two time points. We ranked gene expressions and identified genetic patterns and networks across these diverse isolates that reveal an emphasis on carbon metabolism, fatty acid synthesis, transport, cell wall structure, and stress-related gene functions during growth in CSF. The most highly expressed yeast genes in human CSF included those known to be associated with survival or virulence and highlighted several genes encoding hypothetical proteins. From that group, a gene encoding the *CMP1* putative glycoprotein (CNAG_06000) was selected for functional studies. This gene was found to impact the virulence of Cryptococcus in both mice and the CNS rabbit model, in agreement with a recent study also showing a role in virulence. This transcriptional analysis strategy provides a view of regulated yeast genes across genetic backgrounds important for human CNS infection and a relevant resource for the study of cryptococcal genes, pathways, and networks linked to human disease.

## INTRODUCTION

Cryptococcus neoformans is an encapsulated yeast which became a major cause of meningoencephalitis during the AIDS pandemic (1). Even in the era of antiretroviral therapy, C. neoformans causes several hundred thousand infections per year with substantial morbidity and mortality (2). This yeast possesses a propensity for entering and growing within the brain parenchyma and subarachnoid space of immunocompromised hosts, such as AIDS patients and transplant recipients. Fortunately, over the last 2 decades, sophisticated molecular studies applied to this fungal pathogen have helped to define virulence mechanisms and the production of disease. These have been complemented by global transcriptome studies of the genes and networks which are specifically regulated during infection or under specific environmental conditions (3–7). The regulated genes can then be studied for their direct functional impact on disease. However, many of these transcriptional and functional studies were carried out in a single genetic background, the clinical strain H99, which belongs to the VNI lineage of C. neoformans. Our recent transcriptional study of diverse natural isolates revealed that genotypic differences can impact gene expression during culture in diverse media and in an animal model (7). This transcriptional variation between isolates illustrates the importance of studying diverse isolates to appreciate the breadth of the cryptococcal gene expression profiles and to improve prediction of conserved gene responses and networks.

The transcriptional responses of Cryptococcus are dynamic and highly dependent on the immediate signals from the environmental surroundings of the yeast. In fact, we and others have also shown that gene expression profiles are both site and time specific (5, 8, 9). Therefore, we have worked under the hypothesis that in the cryptococcal disease life cycle, there are six major stages or sites of infection/disease: (i) initiation of infection in the lungs; (ii) yeast survival and proliferation within the lung; (iii) dormancy of the yeasts in host granulomas, which has been mimicked by certain *ex vivo* conditions (10); (iv) reactivation from a latent or dormant infection; (v) dissemination through blood, reticuloendothelial tissues, and the blood-brain barrier; and finally, (vi) proliferation of the yeasts in the brain parenchyma and subarachnoid space. Morphological changes of the yeast during infection may also represent a stage(s) in disease, but more knowledge is needed to better define their roles. Gene expression variation at each of these stages may provide clues as to how the fungus adapts and survives during each and highlight dependencies that may be exploited therapeutically.

We have focused our studies on the proliferation stage of C. neoformans within the central nervous system (CNS), since these are the most frequent clinical stage and site at which clinicians encounter C. neoformans and Cryptococcus gattii in the patient. Our previous work demonstrated that we can identify cryptococcal transcriptomes directly from the subarachnoid space of an animal model, the immunosuppressed rabbit (9). This led us to examine genetic features and pathways utilized by Cryptococcus to produce disease and allow yeast survival. We identified highly upregulated genes in the rabbit CSF that include those that are required for virulence in an animal model (11). Importantly, we were able to directly capture cryptococcal transcriptomes during human infections in two patients, and that study allowed us to predict how Cryptococcus responds to environmental cues within the human CNS (3). We expanded on this work in the current study and evaluated isolates from 31 patients with C. neoformans meningoencephalitis to more fully explore how the Cryptococcus genetic background affects gene expression in the CNS. This investigation has broadened our understanding of genotype-specific transcriptomes as well as the genetic responses conserved in all isolates, even across variable durations of human CNS infections. Furthermore, our gene expression analyses have characterized genes with expression at the human subarachnoid site. We compared the expression profiles of cryptococcal isolates from human CSF to their response to the stresses of artificial CSF and within the rabbit subarachnoid space, as well as to growth in rich media. With these transcriptomes, we identified regulated genes that are both novel and previously identified, and this enabled us to further characterize the pathways and functions important for yeast CNS survival and identify markers of cryptococcal disease in humans.

## RESULTS

### Collection and genetic characterization of C. neoformans isolates.

Cryptococcal yeast cells were isolated from cerebrospinal fluid (CSF) collected from patients in two hospitals in Gaborone and Francistown, Botswana, in 2015. Samples represented the initial lumbar puncture of 34 individual patients with neurological symptoms or signs, prior to receiving any antifungal therapies. All patients had advanced HIV infection with CD4 counts of <100 μl. Of the 34 isolates, 31 were typed as C. neoformans and the remaining three as C. gattii (VGIV), also described as Cryptococcus tetragattii (12). The C. gattii samples were excluded from the composite analysis due to limited sample numbers but were included in the analysis of highly expressed genes for comparison with the C. neoformans CSF samples. Approximately 75% of these individual cases (23/31) occurred in males. The mortality was 42% for cryptococcal meningitis at 72 days in this cohort. The range of CSF quantitative yeast counts from patients was from 10^4^ to 10^7^ CFU/ml. Those with higher CSF yeast counts (10^6^ to 10^7^ CFU/ml) generally yielded the most RNA and represented specimens with the highest sequencing read counts.

**FIG 1:**
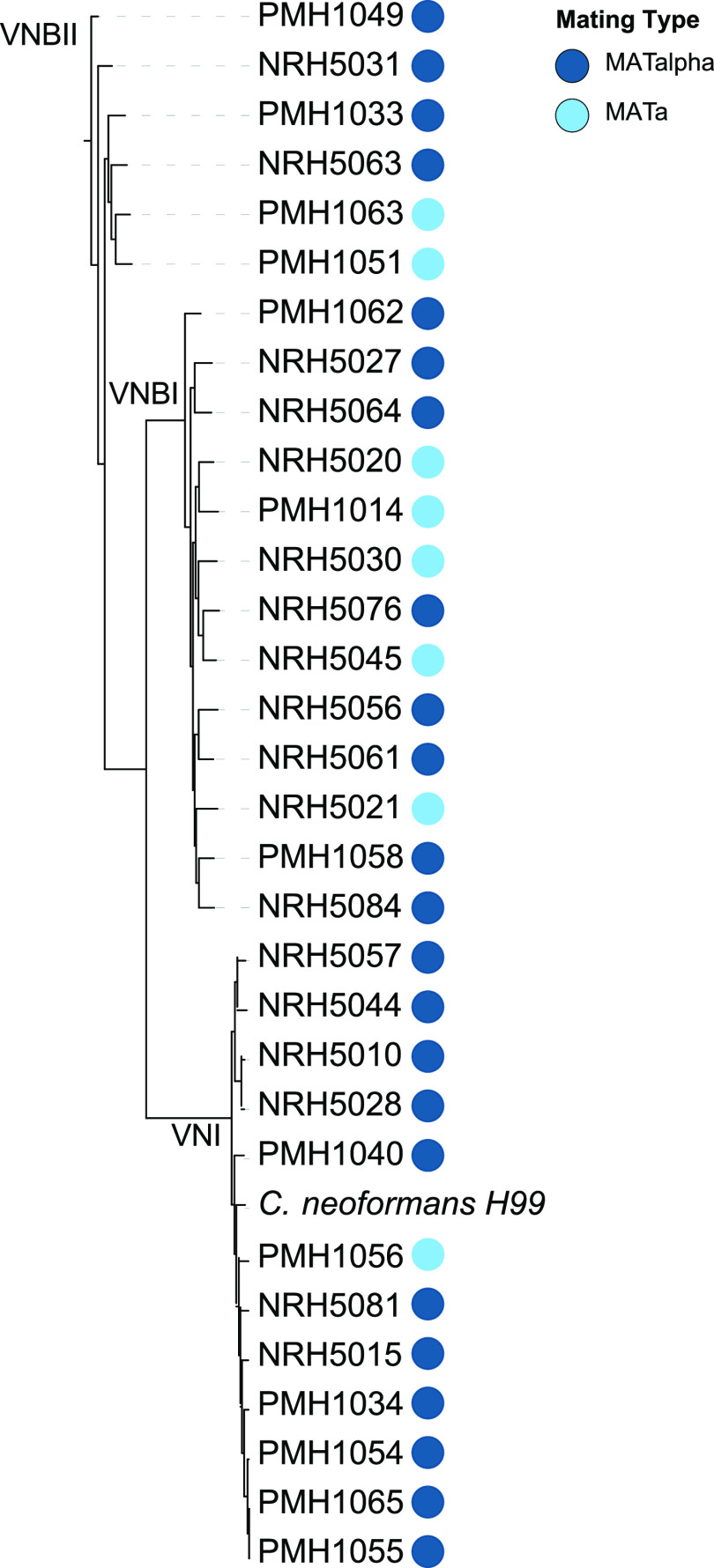
Maximum-likelihood phylogeny of patient isolates. Phylogeny was estimated from 96,943 segregating sites, rooted by VNBII. Isolates separate distinctly into VNI, VNBI, and VNBII, with all lineages having 100% bootstrap support. Colored circles correspond to mating type.

In total, we examined the genetic relationship and gene expression profiles of 31 isolates from different human patients. Genomes were sequenced using Illumina data (Table S1) and reads aligned to the H99 reference genome to call single nucleotide polymorphisms (SNPs). Based on a phylogenetic analysis of 96,943 SNPs, we assigned isolates to either clade VNI, VNBI, or VNBII (Fig. 1). We determined that of these isolates, 1 VNI isolate, 5 VNBI isolates, and 2 VNBII isolates possess the *MAT***a** mating type, while the remaining 23 isolates are *MAT*α. In fact, PMH1056 is only the fourth VNI *MAT***a** isolate reported to date from either patients or the environment worldwide (13). There are 6 isolates that exhibit evidence of aneuploidy, or duplication of large chromosomal segments, based on analysis of normalized read coverage (see Fig. S1 in the supplemental material). Disomy of chromosomes 13 and 4 can be observed in isolates NRH5045 and NRH5076, respectively. Duplications of large regions within chromosomes 4, 5, 9, and 14 are present in NRH5030, NRH5063, PMH1062, and NRH5081, respectively. This level of aneuploidy, or copy number variation, is comparable to what was reported previously for geographically diverse isolates (14). We examined the differentially expressed genes on chromosome 4 and 13 comparing *in vivo* versus yeast peptone dextrose broth (YPD) exposure and did not find any enrichment for known virulence genes or pathways on these chromosomes (Table S4). The apparent advantage of disomy or aneuploidy is therefore not apparent from our gene expression data and not linked to a small number of duplicated genes, as observed with azole heteroresistance and aneuploidy of chromosome 1 containing *AFR1* and *ERG11* (15). Further work will be needed to evaluate the advantage conferred by an extra copy of chromosomes 4 and 13 that could be potentially linked to the differentially expressed genes on these chromosomes.

10.1128/mBio.02313-21.1 TABLE S1 Summary of the conditions and sequencing results of all the RNA-seq samples used in this study, including 33 C. neoformans isolates and 3 C. gattii isolates. List of strains used in the animal studies and list of primers used in this study. Download Table S1, XLSX file, 0.2 MB. Copyright © 2021 Yu et al. 2021 Yu et al. https://creativecommons.org/licenses/by/4.0/ This content is distributed under the terms of the Creative Commons Attribution 4.0 International license.

10.1128/mBio.02313-21.4 TABLE S4 Functional annotations and pathway enrichments of the significantly differential expression genes for three comparisons. “DEGs - in vivo CSF versus YPD” is the list of DEGs from the comparison of *in vivo* CSF versus YPD. “DEGs - hCSF versus rCSF” is the list of DEGs from the comparison of hCSF versus rCSF. “DEGs - rCSF_Day4 versus Day1” is the list of DEGs from the comparison of rCSF on day 4 versus day 1. “GO SLIM & KEGG enrichment” shows the results of the Gene Ontology and KEGG enrichment tests for each upregulated and downregulated DEG in each comparison. “In vivo CSF versus YPD_DEG_chr4&13” is the list of DEGs from the comparison of *in vivo* CSF versus YPD located on the two disomy or aneuploidy chromosomes, chromosome 4 and chromosome 13. “DEG_chr4&13_enrichment” shows results of the GO and KEGG enrichment tests for each up- and downregulated DEG set located on chromosome 4 or 13. Download Table S4, XLSX file, 0.5 MB. Copyright © 2021 Yu et al. 2021 Yu et al. https://creativecommons.org/licenses/by/4.0/ This content is distributed under the terms of the Creative Commons Attribution 4.0 International license.

10.1128/mBio.02313-21.5 FIG S1 Chromosomal copy numbers represented by depth of coverage, normalized by the average genome-wide sequence depth. Isolates displaying whole-chromosome or large partial chromosomal duplications are shown. Download FIG S1, TIF file, 1.4 MB. Copyright © 2021 Yu et al. 2021 Yu et al. https://creativecommons.org/licenses/by/4.0/ This content is distributed under the terms of the Creative Commons Attribution 4.0 International license.

### Highly expressed Cryptococcus genes during infection of human CNS.

The CNS and specifically the subarachnoid space is the most clinically relevant body site of infection. This site represents the stage where individuals generally seek treatment and where the disease becomes life-threatening to the host. Defining the cryptococcal genes that are highly expressed or downregulated in the CNS during infection in the human host can provide insight into the genes and genetic networks specifically activated in the yeast by this hostile environment. These genetic signatures could represent biomarkers of infection progression or as potential targets for therapeutics. We compared gene expression levels across the 31 clinical isolates by sequencing cryptococcal RNA directly from human CSF and ranked the most highly expressed genes among the samples (Table S2). We anticipated that the most highly expressed genes would involve nutrient acquisition, energy production, and cell wall remodeling. This list may serve as a reference for others in the cryptococcal molecular pathogenesis field to examine the potential utilization of their cryptococcal genes or networks in the host. This analysis also allowed us to independently compare and validate our transcriptomic findings with a previous small cohort (3).

10.1128/mBio.02313-21.2 TABLE S2 Gene expression ranking of all 31 good-quality C. neoformans hCSF RNA-seq samples. “Gene expression” shows the expected read counts (RSEM) of all genes for all 31 hCSF samples. “Ranked median” shows values representing the rank of the gene expressed in each hCSF sample. The last column shows the medians of the gene ranks out of all 31 hCSF samples. Download Table S2, XLSX file, 2.5 MB. Copyright © 2021 Yu et al. 2021 Yu et al. https://creativecommons.org/licenses/by/4.0/ This content is distributed under the terms of the Creative Commons Attribution 4.0 International license.

To focus on critical gene expression in the CSF, we focused on the 50 most highly expressed genes (Table 1). Of these 50 genes, at least 7 (*CMP1*, *CIG1*, *ENA1*, *RIM101*, *CDA1*, *CQS1*, and *RCK2*) have previously been associated with virulence or survival within a cryptococcal host model system and at least 2 (*FKS1* and *EF3*) are essential for C. neoformans growth. Notably, multiple cell wall-associated genes are found on this list of genes most highly expressed in the CSF. The cryptococcal cell wall provides foundational yeast cell structure and integrity for the yeast, is critical for the attachment of virulence factors such as the polysaccharide capsule, melanin, and phospholipase, and represents the interface for host interactions. We observed that several genes associated with cell wall metabolism are highly expressed, including 1,3-beta-glucan synthase (*FKS1*), alpha-1,3-glucan synthase, glucan 1,3-beta-glucosidase, and endo-1,3(4)-beta-glucanase. Another cell wall-associated, highly expressed gene, *CDA1*, encodes a major chitin deacetylase that accounts for all of the chitosan produced during vegetative growth to maintain cell integrity and aids in bud separation for the yeast cell (16). *CDA1* is important for the full virulence of a cryptococcal strain and the Cda1 protein is highly antigenic and can be utilized for protective vaccines in mice (17). We also identified two genes that encode candidate mannoproteins, Cmp1 (18) and Mpn10 (19). Finally, Rim101 is a transcription factor important for yeast adaptation to higher pH in part through the remodeling of the cell wall (6). The subarachnoid space is slightly basic but relatively stable, so this transcription factor may be primarily involved in cell wall remodeling. Interestingly, Rim101 was not identified in a transcription factor library screen as being important for brain infection in the mouse (20). However, our results show that *RIM101* and eight genes (*AGS1*, *EBG1*, *ENA1*, *CDA1*, *CFO1*, *CIG1*, *SIT1*, and *FKS1*) associated with Rim101 (6, 21–23) were among the top 50 most highly expressed genes. This highlights the importance of studying genes at the site of infection using different methods and models.

**TABLE 1:** Top 50 expressed genes for all human CSF samples

Category	Gene	Symbol	Description	Median CPM	Secreted proteins	High C. gattii CPM
Cell wall metabolism	CNAG_06000^*a*^	CMP1	Glycoprotein	6,250	N	Y
	CNAG_06508	FKS1	1,3-β-Glucan synthase component FKS1	4,688	N	Y
	CNAG_03120	AGS1	α-1,3-Glucan synthase	4,395	N	Y
	CNAG_05138		Glucan-1,3-β-glucosidase	3,072	N	N
	CNAG_02860	EBG1	Endo-1,3(4)-β-glucanase	1,783	Y	N
	CNAG_00261	MPN10	Putative mannoprotein	1,721	N	N

Extracellular vesicles	CNAG_06241^*a*^	CFO1	Acidic laccase	3,946	N	Y
	CNAG_06347	BLP2	pr4/barwin domain protein	3,885	N	N
	CNAG_01562	BLP4	pr4/barwin domain protein	3,232	Y	Y
	CNAG_00311		3-Hydroxyisobutyryl-CoA hydrolase	2,687	N	N
	CNAG_05799	CDA1	Chitin deacetylase	2,542	Y	Y
	CNAG_02030		Glyoxal oxidase	2,457	Y	N

Fatty acid metabolism	CNAG_01565		Biotin-(acetyl-CoA-carboxylase) ligase	6,470	N	N
	CNAG_01150		Omega-6 fatty acid desaturase (delta-12 desaturase)	4,499	N	Y
	CNAG_04687		Stearoyl-CoA desaturase (delta-9 desaturase)	4,258	N	Y

Glucose metabolism	CNAG_06081		Glucose oxidase	2,296	N	Y
	CNAG_06699	GPD1	Glyceraldehyde-3-phosphate dehydrogenase	1,858	Y	N
	CNAG_07561		6-Phosphogluconate dehydrogenase	1,817	Y	N

Iron uptake	CNAG_01653	CIG1	Cytokine inducing-glycoprotein	9,821	Y	N
	CNAG_00815^*a*^	SIT1	MFS transporter, SIT family	3,740	N	N

Pentose-phosphate pathway	CNAG_01984	TAL1	Transaldolase	2,057	Y	Y

pH response	CNAG_06400	PMA1	Plasma membrane proton efflux P-type ATPase	6,623	N	Y
	CNAG_00531^*a*^	ENA1	Potassium/sodium efflux P-type ATPase	4,484	N	Y
	CNAG_05431^*a*^	RIM101	pH response transcription factor pacC/RIM101	2,300	N	Y

Translational cofactor	CNAG_01117	EF3	Elongation factor 3	7,236	N	Y
	CNAG_06125	TEF1	Elongation factor 1-alpha	6,743	Y	Y
	CNAG_06840		Elongation factor 2	3,260	Y	Y

Other functions	CNAG_06101		ADP, ATP carrier protein	5,543	Y	Y
	CNAG_00456^*a*^	ISP6	Identified spore protein 6	5,440	N	N
	CNAG_06576	CAR1	cAMP-regulated gene 1	4,041	N	N
	CNAG_06267	RDS1	Rds1-like protein	4,041	Y	N
	CNAG_02943		Cytoplasmic protein	2,787	Y	N
	CNAG_04735	MEP1	Extracellular elastinolytic metalloproteinase	2,787	N	Y
	CNAG_00483	ACT1	Actin	2,686	Y	N
	CNAG_00147		Pre-mRNA-processing-splicing factor 8	2,508	N	Y
	CNAG_03012	CQS1	Quorum-sensing-like molecule	2,436	N	N
	CNAG_00091^*a*^		Interaction with SWI/SNF complex	2,434	N	N
	CNAG_04625		Cerevisin	2,230	N	N
	CNAG_03143	HSP	Heat shock protein	2,152	N	N
	CNAG_04904	CHC1	Clathrin heavy chain	2,023	N	N
	CNAG_07538		Calcium/proton exchanger	2,007	N	N
	CNAG_06220		Allergen	1,957	N	N
	CNAG_05750	ATP1	ATP synthase subunit alpha, mitochondrial	1,904	Y	N
	CNAG_01722		Vacuolar protein sorting-associated protein vps13	1,859	N	N
	CNAG_00130	RCK2	CAMK/CAMK1/CAMK1-RCK protein kinase	1,522	N	N

Hypothetical proteins	CNAG_00848		Hypothetical protein	5,928	N	N
	CNAG_02129^*a*^		Hypothetical protein	4,177	N	N
	CNAG_07888		Hypothetical protein	3,176	N	N
	CNAG_00995		Hypothetical protein	2,999	N	N
	CNAG_04105		Hypothetical protein	1,889	N	N

a Gene also identified in a prior comparison of *in vivo* CSF and YPD (3).

Many of these most highly expressed genes are associated with several known important functions required for the survival and virulence of C. neoformans. Three genes, encoding biotin-(acetyl coenzyme A [acetyl-CoA]-carboxylase) ligase, omega-6 fatty acid desaturase (delta-12 desaturase), and stearoyl-CoA desaturase (delta-9 desaturase), are involved in fatty acid metabolism. We had previously shown that *FAS1* (fatty acid synthase) is required for basic cryptococcal survival (24), but fatty acid metabolism may be particularly important in the CNS environment. Furthermore, we showed previously that the intact glycolytic pathway is important for survival of C. neoformans in the CNS of the rabbit (25). Another set of genes, including those for glucose oxidase, glyceraldehyde-3-phosphate dehydrogenase, 6-phosphogluconate dehydrogenase, decarboxylating 1, are associated with glucose metabolism, which is clearly a metabolic focus for C. neoformans under host stress in the human CNS (20).

In addition, genes important for pH maintenance and iron uptake were highly expressed. Two genes encoding P-type ATPases, *ENA1* and *PMA1*, are regulated in response to ionic homeostasis (26). An *ena1Δ* mutant is avirulent and rapidly cleared not only from mice (27) but also from rabbit CSF (28). Interestingly, *ENA1* is essential for simply surviving in *ex vivo* CSF as well as within the subarachnoid space and thus is a uniquely pivotal gene for CNS survival. Another pathway, the cyclic-AMP/protein kinase A (cAMP/PKA) signal transduction pathway, is critical to respond to host conditions, such as by capsule production and increased iron uptake, as well as for growth inside the infected host (29). In the human, Cryptococcus appears to also sense the low-iron environment within the subarachnoid space. For instance, two highly expressed genes include that for the *PKA1* signaling pathway cytokine-inducing glycoprotein, *CIG1*, that is associated with heme and iron uptake (21, 30) and *SIT1*, encoding a siderophore transporter (31). These genes are both connected to the Rim101 and cAMP/PKA signaling pathways that are integrated into the known genetic virulence composite of C. neoformans (6, 29).

Other notable functions include several proteins linked to environmental responses or interactions. For instance, other highly expressed genes, including *CFO1*, *BLP2*, *BLP4*, *CDA1*, and the glyoxal oxidase gene, also encode membrane-bound proteins which have been discovered in extracellular vesicles produced by this yeast and shown to participate in the export of virulence factors (32). The quorum-sensing gene *CQS1/QSP1* is required for full virulence and is also highly expressed (33). A predicted heat shock protein (CNAG_03143) has been found to be upregulated in inositol, a known stimulator for promoting Cryptococcus penetration into the brain (34), and is also highly expressed in CSF. Lastly, there are many highly expressed C. neoformans genes in human CSF (hCSF) without a predicted function, which supports further study for their role in pathogenesis and potentially as drug targets. Taken together, these observations show that Cryptococcus genes highly expressed in the CNS are important to the yeast.

We also assessed the transcriptome sequencing (RNA-seq) expression profiles of three C. gattii VGIV isolates to identify their highly expressed genes (Table S3). After comparing these two rank lists of highly expressed gene with orthologous gene mapping, we found that of these top 50 highly expressed genes of the VGIV isolates, 19 genes (38%) overlap the top 50 C. neoformans (Table 1) and 16 additional genes (29%) are in the top 120 C. neoformans genes. Considering the known high variances of the gene expressions in individual human CSF samples and the limit of the orthologous gene identifications, this result suggests that many of these most highly expressed genes in human CSF are consistently highly expressed across multiple isolates and species.

10.1128/mBio.02313-21.3 TABLE S3 List of genes sorted by expression level for each C. gattii hCSF sample. Genes marked with colors are the highly expressed genes overlapping between C. neoformans and C. gattii isolates. The NRH5051 column is grey because this sample was removed due to the low total read count. Download Table S3, XLSX file, 0.4 MB. Copyright © 2021 Yu et al. 2021 Yu et al. https://creativecommons.org/licenses/by/4.0/ This content is distributed under the terms of the Creative Commons Attribution 4.0 International license.

To complement our analysis of quantitative expression rankings, we also attempted to provide a global view of gene functions that appear important to the yeast under CNS stress. Therefore, we carried out a gene set enrichment analysis (GSEA) to identify functional categories enriched in genes that are highly expressed in CSF. This analysis showed that the most significantly enriched functional terms included those involved in metabolism of carbohydrates and lipids and ion binding and metabolism (Table 2), similar functions to those highlighted in the analysis of individual most highly expressed genes.

**TABLE 2:** Gene set enrichment analysis of ranked highly expressed genes in human CSF

GO category	GO term^*a*^	FDR *P* value
Biological process	Glycolysis (GO:0006096)	0.001
	Cation transport (GO:0006812)	0.024
	Carbohydrate metabolic process (GO:0005975)	0.048
	Lipid metabolic process (GO:0006629)	0.040
	Signal transduction (GO:0007165)	0.035

Molecular function	GTPase activity (GO:0003924)	0.013
	Magnesium ion binding (GO:0000287)	0.014
	Structural molecule activity (GO:0005198)	0.022
	Calcium ion binding (GO:0005509)	0.033
	Structural constituent of ribosome (GO:0003735)	0.034
	ATPase activity (GO:0016887)	0.039
	Metal ion binding (GO:0046872)	0.042

Cellular component	Endoplasmic reticulum (GO:0005783)	0.005
	Ribosome (GO:0005840)	0.006
	Intracellular (GO:0005622)	0.014
	Cytoplasm (GO:0005737)	0.032

a GO terms with FDR *P* values of ≤0.05 are listed.

### Comparisons of gene expression profiles across conditions.

**FIG 2:**
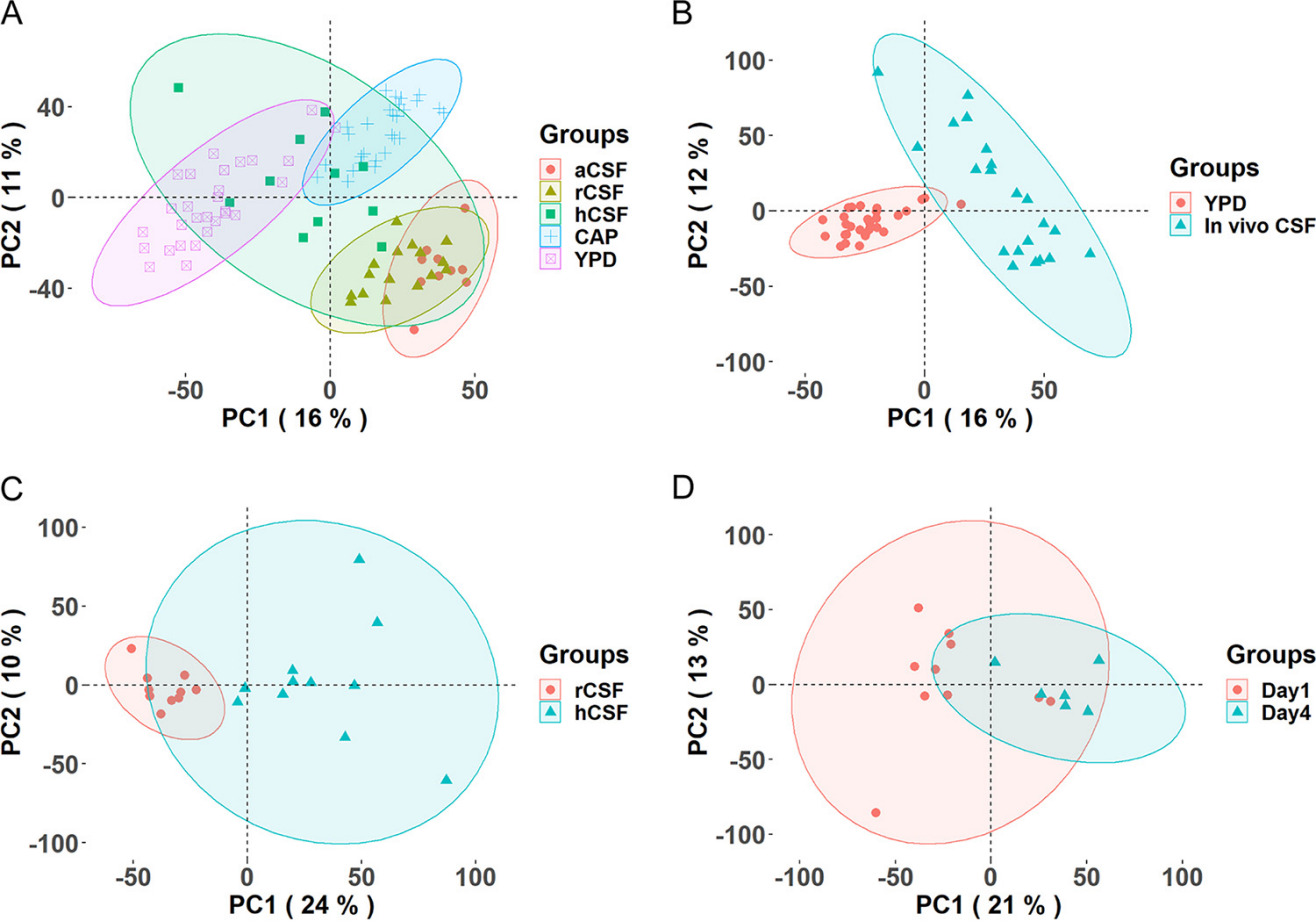
PCA of the condition comparisons. PCA of the gene expression profiles was done across all conditions, including artificial CSF (aCSF), rabbit CSF (rCSF), human CSF (hCSF), capsule-inducing medium (CAP), and yeast peptone dextrose broth (YPD) (A); between the *in vivo* CSF (human CSF and rabbit CSF) and YPD samples (B); between the rabbit CSF and human CSF samples (C); and between the rabbit CSF samples on day 4 and day 1 (D).

In our previous study comparing gene expression in environmental isolates to clinical isolates (7), we showed that while lineage assignment or genotype was a major contributor to gene expression variation and worthy of further study, the growth condition was a much larger contributing factor than lineage to expression patterns. Therefore, to provide a comparator for gene expression in human CSF, we characterized the differences in gene expression between yeasts isolated directly from hCSF to those grown under other relevant *in vivo* and *in vitro* conditions. We compared expression profiles for the hCSF condition to four additional conditions: rabbit CSF (rCSF), artificial CSF (aCSF), capsule-inducing medium (CAP), and rich medium (YPD). After filtering out low-quality samples (fewer than 6,000 genes detected), we analyzed 10 aCSF, 11 hCSF, 16 rCSF, 28 CAP, and 29 YPD samples for these condition comparisons. A principal-component analysis (PCA) of gene expression shows separation between samples from different conditions (Fig. 2A). For example, rabbit CSF samples and artificial CSF samples appear closely grouped in their expression patterns but are clearly separated from those grown in YPD and CAP. As expected, the heterogenous human CSF samples showed the most divergent expression profiles across samples.

When strains from *in vivo* CSF conditions (human and rabbit) are compared to those grown in YPD, the *in vivo* CSF strains form a separate and more dispersed cluster than the more uniform YPD samples (Fig. 2B). The 1,079 genes differentially expressed (*P* < 0.001) between the *in vivo* CSF conditions and YPD showed significant enrichment for functions including transport, carbohydrate metabolism, fatty acid metabolism, and lipid processing (Table 3). This reflects the particularly high expression levels of these pathways across hCSF samples (Table 2). Differentially expressed genes between the *in vivo* CSF conditions and YPD also include 76 genes responsive to stress, 30 transcription factor genes, and 25 ion transporter genes, with the majority of these (∼86%) being upregulated within the subarachnoid space. We also found 10 genes specifically associated with oxidative and nitrosative stress both up- and downregulated between conditions (Table S4).

**TABLE 3:** Significant enrichment of the Slim Gene Ontology and KEGG pathway for the differential expression gene sets

DEG comparison	GO term/KEGG pathway^*a*^	FDR *P* value
*In vivo* CSF vs YPD upregulated	Transmembrane transport (GO:0055085)	8.28E−18
	Transport (GO:0006810)	3.49E−11
	Fatty acid degradation (ec00071_KEGG)	3.15E−06
	Biological process (GO:0008150)	2.62E−03
	Valine, leucine, and isoleucine degradation (ec00280_KEGG)	1.22E−07
	Carbohydrate metabolic process (GO:0005975)	8.70E−04
	Geraniol degradation (ec00281_KEGG)	3.15E−03
	α-Linolenic acid metabolism (ec00592_KEGG)	3.15E−03
	Metabolism of xenobiotics by cytochrome P450 (ec00980_KEGG)	1.23E−02
	Fatty acid elongation (ec00062_KEGG)	2.08E−02
	Lysine degradation (ec00310_KEGG)	2.54E−02
	β-Alanine metabolism (ec00410_KEGG)	3.76E−02
		

*In vivo* CSF vs YPD downregulated	Lipid metabolic process (GO:0006629)	8.29E−04
	Generation of precursor metabolites and energy (GO:0006091)	8.98E−04
	Butanoate metabolism (ec00650_KEGG)	2.96E−02
		

hCSF vs rCSF upregulated	Carbohydrate metabolic process (GO:0005975)	6.68E−11
	Citrate cycle (TCA cycle) (ec00020_KEGG)	4.89E−04
	Carbon fixation in photosynthetic organisms (ec00710_KEGG)	6.14E−03
	Generation of precursor metabolites and energy (GO:0006091)	1.59E−02
	Pentose phosphate pathway (ec00030_KEGG)	9.11E−05
	Glycolysis/gluconeogenesis (ec00010_KEGG)	1.42E−04
	Biosynthesis of antibiotics (ec01130_KEGG)	2.28E−03
	Small molecule metabolic process (GO:0044281)	2.28E−03
	Glutathione metabolism (ec00480_KEGG)	4.77E−03
	Starch and sucrose metabolism (ec00500_KEGG)	1.25E−02
	Cofactor metabolic process (GO:0051186)	1.25E−02

a GO terms and KEGG pathways with FDR *P* values of ≤0.05 are listed.

Comparing expression profiles of the two *in vivo* CSF conditions reveals clear separation of human and rabbit CSF samples (Fig. 2C) despite higher variation within the hCSF cohort. The length of time that Cryptococcus was in the hCSF is undefined as patients randomly enter the hospital with an established CNS infection. For C. neoformans in the human CSF, carbohydrate metabolism appears strongly enriched, with genes involved in the tricarboxylic acid (TCA) cycle, the pentose phosphate pathway, glycolysis, and complex sugar metabolism being upregulated (Table 3). There were also 70 genes responsive to stress that were differentially regulated between hCSF and rCSF samples, along with 30 transcription factors and 20 ion transporters which were mainly induced in the hCSF. Protein kinase genes were found to be upregulated in hCSF twice as often as in rCSF, and there were few differentially regulated genes which were involved in oxidative or nitrosative functions (Table S4).

To provide a perspective on the variability of cryptococcal expression within the subarachnoid space over time and to examine the rapidity of dynamic transcriptional changes in the *in vivo* CSF, we performed a longitudinal rabbit study over 3 days. The longitudinal rabbit CSF data allow the comparison of strains grown *in vivo* for two time periods (day 1 and day 4). We chose day 1 to understand the initial stress of yeasts entering the subarachnoid space and day 4 as the time when the quantity of yeasts in the CSF seems to stabilize and is similar to that found in human CSF. Samples isolated from rCSF at day 1 and day 4 postinoculation cluster based on time within the rabbit host. Samples collected at day 4 cluster tightly compared to those collected at day 1, highlighting a converging rCSF expression profile over exposure time at the body site (Fig. 2D). These results highlight the importance of nutrient acquisition, carbohydrate metabolism, and response to stress as well as the transitioning of the yeast cells to the host environment. There were 93 genes differentially expressed (*P* < 0.001) over 72 h, and of these, 41 genes are annotated as hypothetical proteins, 10 genes are annotated as transporters, 4 are implicated in carbohydrate metabolism, 5 encode protein processing genes, and 7 are responsive to stress (Table S4). This highlights the dynamic transition required of the yeast to maintain itself in the subarachnoid space, and this adaptation likely involves nutrient acquisition, energy production, and stress pathway activations.

### Regulation of pathways during *in vivo* growth.

**FIG 3:**
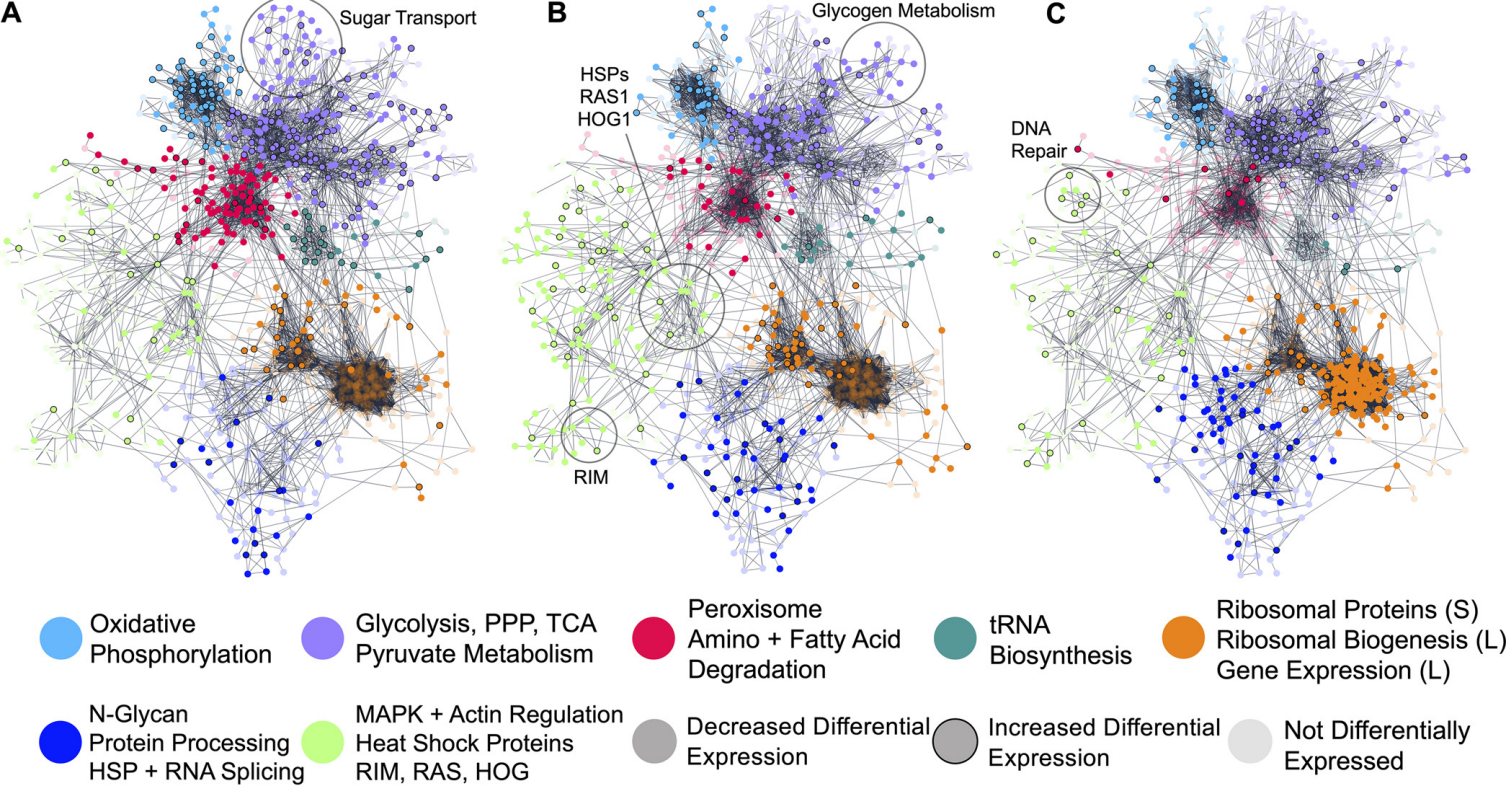
Protein-protein interaction network displaying regulatory modules of significantly differentially expressed genes. Genes differentially expressed between condition comparisons are represented by opaque nodes. Genes which are not differentially expressed between conditions are represented by translucent nodes. (A) YPD versus human plus rabbit CSF. Nodes outlined in black denote genes displaying increased expression in YPD compared to both human and rabbit CSF. (B) Rabbit versus human CSF. Nodes outlined in black denote genes displaying increased expression in rabbit CSF compared to human CSF. (C) Day 1 versus day 4 CSF. Nodes outlined in black denote genes displaying increased expression on day 4 compared to day 1 within rabbit CSF.

To identify specific pathways and functional modules alternately regulated between these specific environments, we then performed module analysis with genes significantly and differentially expressed between nutrient-rich (YPD) and stress (*in vivo* CSF) conditions. Module analysis found that isolates grown in YPD upregulate sterol metabolism, glycolysis, the TCA cycle, and oxidative phosphorylation compared to C. neoformans isolated from the limited-nutrient (stress) environments of *in vivo* CSF and the subarachnoid space. However, within *in vivo* CSF, C. neoformans upregulates fatty acid degradation and sugar transport, indicative of the increased nutrient transport required in this nutrient limited environment (Fig. 3A [YPD versus *in vivo* CSF]). Comparison of isolates from rabbit versus human CSF *in vivo* revealed that C. neoformans upregulates pathways involved in mitogen-activated protein kinase (MAPK) signaling, actin regulation, and heat shock response within the rabbit model, perhaps reflective of the suddenly increased body temperatures of rabbits and acute nature of the rabbit infection compared to human disease. Isolates from human CSF upregulate the RIM pathway (*RIM101*, *RIM20*, *RIM13*, *PALC*, and *SNF7*), RAS1 signaling, HOG1 signaling, and glycogen metabolism (Fig. 3B). This finding suggests that *in vivo* responses are influenced by pH, temperature, ion homeostasis, and other external stressors, including energy capabilities. In C. neoformans isolated serially from rabbit CSF, there is a marked downregulation of genes involved in gene expression, protein processing, and ribosomal biogenesis at day 4 compared to the initial infection (day 1). This is suggestive of yeast growth arrest as the yeast attempts to equilibrate and adapt to its new site of infection (Fig. 3C).

### Functional impact of the highly expressed gene *CMP1*.

**FIG 4:**
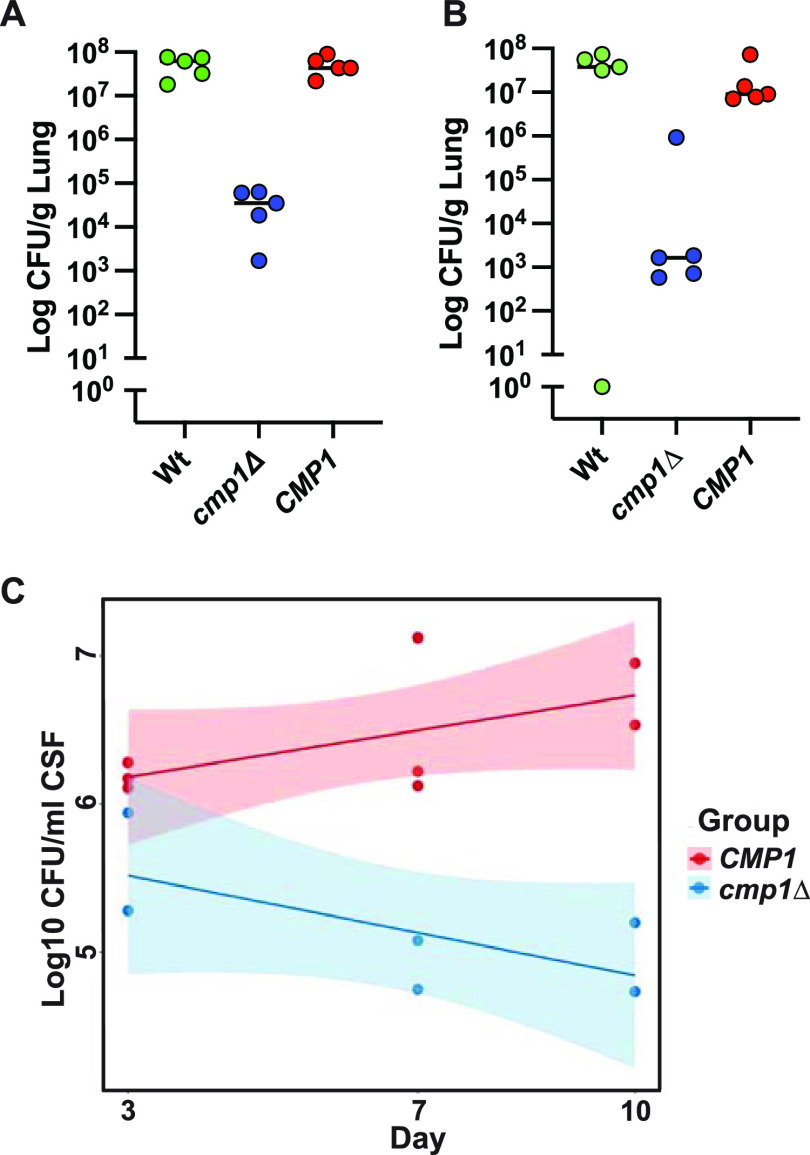
Effect of *CMP1* on virulence in mice and rabbits. CD-1 mice were infected with 5 × 10^4^ CFU/ml (A and B). At 14 days of infection, the lungs were assessed for fungal burden in 5 male mice (A) and 5 female mice (B). The *cmp1Δ* mutant had reduced fungal burden in both male and female mice compared to the reconstituted *CMP1* (*cmp1Δ::CMP1*) strain (*P* = 0.0079 for both comparisons). (C) The results from 3 male New Zealand White rabbits with fungal burden assessed on day 3, day 7, and day 10 postinoculation showed the significant effect of *CMP1*. The *P* value from the repeated-measures analysis is 0.00021.

To analyze highly expressed Cryptococcus genes of unknown function in the human subarachnoid space, we selected to test one of the most highly expressed genes for its impact on virulence. This gene (*CMP1*, CNAG_06000) was recently characterized a putative mannoprotein, and loss of this gene resulted in an attenuated cryptococcal strain in mice (18). In our study of a *cmp1Δ* mutant, we found that loss of this gene, in both male and female CD-1 mice, resulted in a reduced fungal burden compared to the reconstituted strain (*P* < 0.01) (Fig. 4A and B) and confirmed others’ results (18). However, as a mouse inhalation model reflects a pulmonary infection more than a CNS infection, we also examined the fungal burden of the mutant in the CSF of rabbits. In this rabbit experiment, the *cmp1Δ* strain is also reduced in its ability to survive in CSF, suggesting that this gene is important for yeast persistence in the CSF (Fig. 4C). Thus, a highly regulated yeast gene initially identified in hCSF during cryptococcal meningitis was demonstrated to be an important gene for CNS survival.

## DISCUSSION

The diversity of natural isolates that cause cryptococcal pathogenesis needs to be more widely considered in individual gene studies. Here, we present the transcriptional responses during *in vivo* growth and control conditions for a diverse set of isolates spanning the VNI, VNBI, and VNBII genotypes. While in many countries, clinical isolates of C. neoformans are often dominated by the VNI genotype, along with the less common VNII genotype (13, 14, 35), isolates of the VNBI and VNBII genotype are commonly reported from southern Africa and more rarely in other countries (13, 14, 36). While isolates of both mating types are represented in this study, we identified only 8 *MAT***a** isolates, including an unusual *MAT***a** VNl isolate. By comparing gene expression in CSF across samples representing three of the four major lineages, we highlighted the major signatures of gene expression independent of lineage.

Through transcriptional pathway analysis, we characterized the major differences between conditions and found metabolic pathways including glycolysis, the TCA cycle, and oxidative phosphorylation to be highly upregulated in nutrient-rich conditions, consistent with increased carbohydrate metabolism in YPD (7). Yeasts isolated from human CSF upregulate sugar transport, highlighting the requirement of carbohydrate transport for metabolism and survival in the limited-nutrient host environment. This focus on carbohydrate transport is a hallmark of the transcriptional response of C. neoformans to the limited-nutrient environment of the lung and CSF in animal models (5, 7, 25, 37). Furthermore, C. neoformans responds specifically to human CSF by upregulating the *RIM101* pathway, involved in pH-mediated host adaptation and immune evasion (6, 22, 23, 37). We also found differential regulation of *HOG1*, central to stress response and capsule regulation (38, 39), and *RAS1*, required for thermotolerance and morphogenesis in human CSF (37, 40). This is consistent with the upregulation of *RAS1* in Candida albicans during mock bloodstream infections (41). In contrast, during persistent infection of the rabbit CSF, C. neoformans responds with metabolic muting and a reduction in protein processing and ribosomal biogenesis, perhaps indicative of growth arrest that is adaptive to a limited-nutrient environment. This finding would be consistent with observations of metabolic dormancy in subpopulations of C. neoformans in response to extended nutrient limitation (10).

Both our analysis of highly expressed genes in the human CSF and prior data support the idea that C. neoformans is upregulating carbon metabolism and specifically glycolysis. We previously evaluated the functional importance of glycolysis compared to gluconeogenesis for Cryptococcus at multiple body sites. For instance, blocking the ability of Cryptococcus to use 2- and 3-carbon substrates for gluconeogenesis in a phosphoenolpyruvate carboxykinase mutant (*pck1Δ*) revealed a critical requirement of gluconeogenesis for yeast survival in the lung; however, *PCK1* does not appear to be important for growth in the CSF of rabbits. In contrast, the enzyme pyruvate kinase, encoded by *PYK1*, which is required for glycolysis, is essential for survival in the subarachnoid space (25). Our expression and functional studies have clearly identified the importance of yeast carbon metabolism in the CSF and specifically, glycolysis, for energy production during growth in the subarachnoid space.

In comparing data from human samples and animal studies, our transcriptomic data highlight differences between high levels of gene expression and pathobiological function. We detected major differences in pathways involved in pH, temperature, and ion homeostasis, suggesting differences in these factors between the human and rabbit samples. Notably, there are two highly expressed cryptococcal genes in human CSF which have dramatic differences between expression and function. *ENA1*, a potassium/sodium efflux P-type ATPase, is highly expressed in the human CSF. The *ena1Δ* mutant does not survive well in *ex vivo* human CSF, mice, and the subarachnoid space of rabbits (27, 28). On the other hand, *CQS1* (also known as *QSP1*), a quorum-sensing gene, is highly upregulated in human CSF, which suggests that yeast cells may sense other yeast cells. However, in the rabbit subarachnoid space, the *qsp1*Δ mutant survives similarly to the wild-type yeast (33).

In examining highly expressed genes in human CSF, we sought to evaluate if high expression could be a factor that would enrich for genes essential for the ability of diverse C. neoformans isolates to cause disease. We focused our further study on the highly expressed hypothetical glycoprotein gene (CNAG_06000 or *CMP1* [cryptococcal mannoprotein 1]) shown in a recent study to be a downstream target of the C. neoformans F-box protein, Fbp1, and to encode a mannoprotein (18). Glycoproteins are known to have low content in the cryptococcal capsule but possess high immunogenicity. *CMP1* was found to be linked to capsule production, expressed in all stages of cryptococcal development, protected yeast cells against complement and intracellular macrophage growth retardation, and was important for cryptococcal virulence in mice (18). Surface proteins and other proteins that alter the cell wall can affect the immune response of the host to the fungus. We confirmed that *CMP1* is not only important to virulence of C. neoformans in the mouse but also important to survival of the yeast in the subarachnoid space of rabbits. These findings support that the genes identified based on high-level CSF expression may be enriched and linked to disease production in the human host by validating the importance of *CMP1* in two other mammalian hosts. This also further suggests the pathobiological importance of mannoproteins with glycosylphosphatidylinositol (GPI) anchors in C. neoformans such as Cmp1. For instance, an inhibitor of the Gwt1 enzyme, APX2039, has extremely potent anticryptococcal activity both *in vitro* and *in vivo* (42). APX2039 blocks the localization of GPI-anchored cell wall mannoproteins. Mannoproteins are likely rich targets for development of potent antifungal compounds.

Capturing the cryptococcal transcriptome directly at the human site of infection allows investigators a window into how the yeast adapts in the human to specific body sites. This approach identified genes previously categorized as important for CNS infection and, notably, also identified genes of unknown function that warrant further study. These results also highlight certain pathways of structure and metabolism that are critical for C. neoformans disease. We have shown that the variability we observe across transcriptomes is affected by the genetic background of the different isolates and we expect that the incubation time within the host is also a major determinant of gene expression. However, we do not have information on when patients were infected to estimate the length of time Cryptococcus was in the patient CSF. Despite this, we have identified a core set of Cryptococcus genes that are highly expressed in human CSF across diverse isolates and presumably infection stages. By capturing these expression profiles, we feel that C. neoformans is talking about its dynamic adaptability in the CNS to cause disease, and it is now our job to listen.

## MATERIALS AND METHODS

### Human subjects.

Human subject research was approved by the Duke University Medical Center Institutional Review Board under protocol Pro00029982.

### Sample preparation and growth conditions.

Descriptions of the clinical C. neoformans strains and conditions examined in this study are listed in Table S1. The clinical yeast isolates were collected directly from the cerebrospinal fluid (CSF) from individual patients with advanced HIV infections and low CD4 counts (<100 cells/μl) and cryptococcal meningitis from two hospitals in Botswana (Princess Marina Hospital in Gaborone and Nyangabgwe Referral Hospital in Francistown). In total, 31 Cryptococcus neoformans isolates were collected from patient CSF samples and were categorized by lineage as 12 VNI, 13 VNBI, and 6 VNBII isolates (Fig. 1). RNA-seq profiles were obtained for each isolate.

RNA was isolated from five additional conditions for a subset of the isolates. (i) The first was artificial CSF (aCSF) with 1 × 10^8^ CFU/ml of yeast cells incubated in aCSF at 37°C for 24 h. Artificial CSF was prepared as described in reference 43. Yeast cells were harvested by centrifugation at 1,932 × *g* and stored at −80°C. (ii) Samples were collected from rabbit CSF (rCSF). CSF yeast cells (1 × 10^9^) were inoculated intracisternally into 2- to 3-kg New Zealand White rabbits. Rabbits received 5 mg/kg hydrocortisone acetate intramuscularly 1 day prior to inoculation and for the duration of the experiment. Each yeast strain was inoculated into three individual rabbits. rCSF was withdrawn (1 to 2 ml) after 24 and 96 h of infection in the rabbit subarachnoid space. rCSF samples from each animal containing the same strain were pooled and centrifuged to pellet the cells, and the cell pellets were stored at −80°C. (iii) Yeast cells (1 × 10^5^ CFU/ml) were grown for 24 h in yeast peptone dextrose broth (YPD), centrifuged to collect the cell pellet, and stored at −80°C. (iv) Capsule-inducing medium (CAP) was prepared using diluted Sabouraud broth in 50 mM MOPS (morpholinepropanesulfonic acid; pH 7.3) as reported in reference 44. (v) One to five milliliters of human CSF (hCSF) containing approximately 10^4^ to 10^7^ CFU of yeasts per ml of CSF was directly withdrawn with a lumbar puncture as standard of care, and CSF remaining after clinical tests was utilized. The hCSF samples were centrifuged, and the cell pellets were stored at −80°C until RNA isolation was performed. In addition to the 31 C. neoformans isolates, RNA-seq was performed for three patient isolates that were subsequently identified as Cryptococcus gattii VGIV (NRH5051, PMH1041, and PMH1053; accessible via PRJNA715187) and therefore excluded from the downstream analysis. Both Duke and Botswana institutional review board (IRB) approvals supported this study.

Strains used for the animal studies and the primer sequences used are listed in Table S1. KN99α (CM026), used as the wild-type strain, and the *cmp1Δ* mutant were obtained from a genome-wide Cryptococcus deletion library (45). The reconstituted *CMP1* strain was generated in this study. Three PCR products were prepared: the *CMP1* locus containing the 5′ flanking sequence, the gene (AD2332/AD2333), and 3′ flanking sequence; the neomycin (NEO) drug-resistance cassette (AD2334/AD2335) amplified from pJAF1 (46); and additional 3′ flanking sequence (AD2336/AD2337). These PCR fragments were fused by overlap PCR using primers AD2296 and AD2297 (47). The PCR product was introduced into the *cmp1Δ* strain by biolistic transformation as previously described (48) and confirmed using primers AD2300 to AD2305.

### Genome sequencing and phylogenetic inference.

Whole-genome sequence was generated in a previous study (13) or was newly generated for three isolates (NRH5081, NRH5084, and NRH5076). For these three isolates, libraries were constructed from genomic DNA using the NEBNext Ultra II protocol. These libraries were sequenced on an Illumina HiSeqX system to generate paired 150-base reads. Sequence reads were aligned to the Cryptococcus neoformans var. *grubii* H99 reference genome (GCA_000149245.3) with BWA-MEM v0.7.17 (49), and variants were called with a GATK v4.1.4.1 (50) variant-calling pipeline developed for fungal genomes (https://github.com/broadinstitute/fungal-wdl/). A maximum-likelihood phylogeny was estimated using 96,943 segregating SNP sites present in one or more isolates and ambiguous in a maximum of 10% of isolates with RAxML v8.2.12 with the GTRCAT model and rapid bootstrapping (51). Chromosomal coverage was determined with funpipe v0.1.0 (https://github.com/broadinstitute/funpipe).

### RNA extraction and sequencing.

Sterile glass beads (1 to 3 mM) were added to the cell pellet prior to freezing or immediately after lyophilization. For RNA extraction, the frozen pellets were lyophilized and vortexed to a fine powder. The yeast cells were lysed in 1 ml of TRIzol (Invitrogen) followed by incubation at room temperature for 5 min. Then, 200 μl of chloroform was added, and the tubes were shaken for 30 s followed by incubation for 3 min at room temperature. The samples were centrifuged at 9,600 × *g* for 15 min. The aqueous phase was collected and mixed with an equal volume of 80% ethanol and immediately applied to a column from the Qiagen RNeasy minikit. The column was then centrifuged at 16,200 × *g* for 1 min. The remaining steps for RNA isolation were performed following the manufacturer’s guidelines (Qiagen).

Libraries were constructed from total RNA using two methods. The samples from YPD and CAP conditions were adapted using the Illumina TruSeq protocol and sequenced on a HiSeq 2500 system to generate paired 101-base reads. All *in vivo* (human and rabbit) and artificial CSF conditions were adapted using the TagSeq protocol (52) in which rRNA was depleted using the RiboZero yeast reagent. Human CSF samples were processed as a batch and sequenced using a HiSeq 2500 system to generate paired 93-base reads. The rCSF and aCSF samples were processed as a batch and sequenced on a NextSeq system to generate paired 75-base reads. Reads from TagSeq libraries were initially processed to remove the inline adapters. After quality filtering and adaptor trimming by Cutadapt (v1.12) (53), the reads were aligned using STAR (v2.5.3a) (54) to the gene set of C. neoformans var. *grubii* H99 (CNA3) (55), excluding noncoding RNAs and mitochondrial genes. After mapping to the H99 genome, at least 19 million paired aligned reads were recovered from each of these RNA-seq libraries. Next, the read counts for each gene were estimated with RSEM (v1.2.31) (56). The raw counts were converted to counts per million (CPM) and then normalized by adjusting with the effective library size via the calcNormFactors function implemented in the R package edgeR (v.3.26.8) (57). For VGIV isolates, the reads were mapped to the C. gattii IND107 genome (GCA_000835755.1), following the above process to evaluate the gene expression profiles. NRH5051 was removed from the analysis of most highly expressed genes due to the very low read count. The median of the gene expression rank was then calculated from the other two samples.

To identify the most highly expressed C. neoformans genes in human CSF, we first sorted all genes based on the normalized expression levels for each human CSF sample. We then calculated the median of the rank number for each gene across all human CSF samples. The rank of all gene expressions was determined by sorting the median rank number of the genes.

To identify enriched functional pathways for the most highly expressed genes, we performed gene set enrichment analysis (GSEA) (58) by applying the GSEAPreranked (https://gsea-msigdb.github.io/gseapreranked-gpmodule/v6/index.html) (v6.0.12) tool on a predefined ranked list of the genes. The ranked gene list was determined by aggregating the sorted gene list of all human CSF samples using the aggregateRanks function in the R package RobustRankAggreg (v.1.1) (59). The rank aggregation method was the default RRA algorithm. The aggregated rank combined with the Cryptococcus H99 Gene Ontology (GO) terms from vEUpathDb (accessed August 2020) (60) were used for GSEA. The significantly enriched GO terms for Biological Process, Cellular Component, and Molecular Function were defined by a false discovery rate (FDR) *P* value of <0.05.

### Differential gene expression analysis.

A gene was considered detected if it had an expected read count from RSEM greater than 1, indicating that at least one read mapped to the gene. Samples with a detected gene count of more than 6,000 were selected for differential expression gene analysis. We compared three condition groups, including *in vivo* (hCSF and rCSF) versus YPD, hCSF versus rCSF, and rCSF (1 day) versus rCSF (4 day). The differentially expressed genes (DEGs) between the three condition groups were determined by implementing the negative binomial generalized linear models with Fisher’s exact tests (exactTest functions in the edgeR package) (57) at the FDR *P* value cutoff of less than 0.01. PCA plots were constructed using the R package factoextra (v.1.0.7) (61). To assess the enriched functional pathways for each DEG gene set, the pathway enrichment analysis was conducted for the GO terms and the KEGG pathway for C. neoformans strain H99 via the FungiDB Enrichment Analysis tool (accessed August 2020 for all analyses except for GO:0006950, for which it was accessed 21 July 2021) (60). This enrichment test was carried out using Fisher’s exact test with the background defined as all genes from the H99 genome. *P* values were corrected for multiple testing using the Bonferroni method.

### Animal studies.

All animal-related study procedures were compliant with the Animal Welfare Act, the *Guide for the Care and Use of Laboratory Animal*s (62), and the Duke Institutional Animal Care and Use Committee (IACUC).

### Murine model.

Wild-type (CM026), *cmp1Δ*, and *CMP1* C. neoformans strains were grown in YPD broth at 30°C in a shaking incubator (220 rpm) for 24 h, centrifuged, and washed twice in phosphate-buffered saline (PBS). The cells were resuspended in PBS and quantified using a T4 cell counter (Nexcelom). Equal numbers of female and male CD-1 mice (Charles River Laboratories) were infected with approximately 5 × 10^4^ CFU per mouse via intranasal aspiration while under isoflurane anesthesia. Mice were monitored daily and observed for acute and chronic adverse symptoms. Mice were sacrificed on day 14. The brain and left lung were homogenized in 1 ml PBS for 25 s using two steel beads and a Mini-Beadbeater 16 apparatus (Biospec Products). The homogenized tissues were serially diluted, and 100 μl from each dilution was plated onto YPD containing 100 μg/ml chloramphenicol. The plates were incubated for 3 days at 30°C. Colonies were counted, and the tissue burden (CFU per gram of tissue) was determined. Fungal burden data were log_10_ transformed and evaluated using *t* tests for unpaired means (Prism software, v9.1.0; GraphPad Software). A *P* value of ≤0.05 was considered statistically significant.

### Rabbit model.

New Zealand White male rabbits weighing 2 to 3 kg were treated with hydrocortisone acetate (2.5 mg/kg) by intramuscular injections daily starting 1 day prior to yeast inoculation. Animals were sedated with ketamine and xylazine and inoculated intracisternally with 0.3 ml of 1 × 10^8^ CFU. For assessing fitness and virulence of Cryptococcus mutants, these animals were infected and cisternal taps were performed on days 3, 7, and 10 followed by enumeration of CFU in the CSF. The time series fungal burden data were then assessed by using a repeated-measures analysis of variance (ANOVA) via the aov (stats v. 4.0.2) function of the R package.

### Pathway analysis.

Network analysis was performed with genes that were significantly differentially expressed in the following comparisons: *in vivo* (human CSF and rabbit CSF) versus YPD, human CSF versus rabbit CSF, and rabbit CSF at day 1 versus rabbit CSF at day 4. ModuleDiscoverer (MODifieRDev v.0.1.3) was employed to identify regulatory modules from significant DEGs (63). Module functions were determined with overrepresentation analysis (hypergeometric test; FDR < 0.0001), and interactions were filtered by STRING interaction scores, with a minimum score of 0.8 required (high confidence) (64).

### Data availability.

Whole-genome sequence data for NRH5081, NRH5084, and NRH5076 can be accessed via PRJNA694643. RNA-seq data are available in the GEO database under accession no. GSE171092, and data for C. gattii VGIV isolates are available at accession no. PRJNA715187.
