# Supplementary figures and images for: Gene Expression of Diverse Cryptococcus Isolates during Infection of the Human Central Nervous System
Source: mBio. 2021 Nov 2;12(6):e02313-21. doi: 10.1128/mBio.02313-21 (PMC8561399; doi:10.1128/mBio.02313-21)

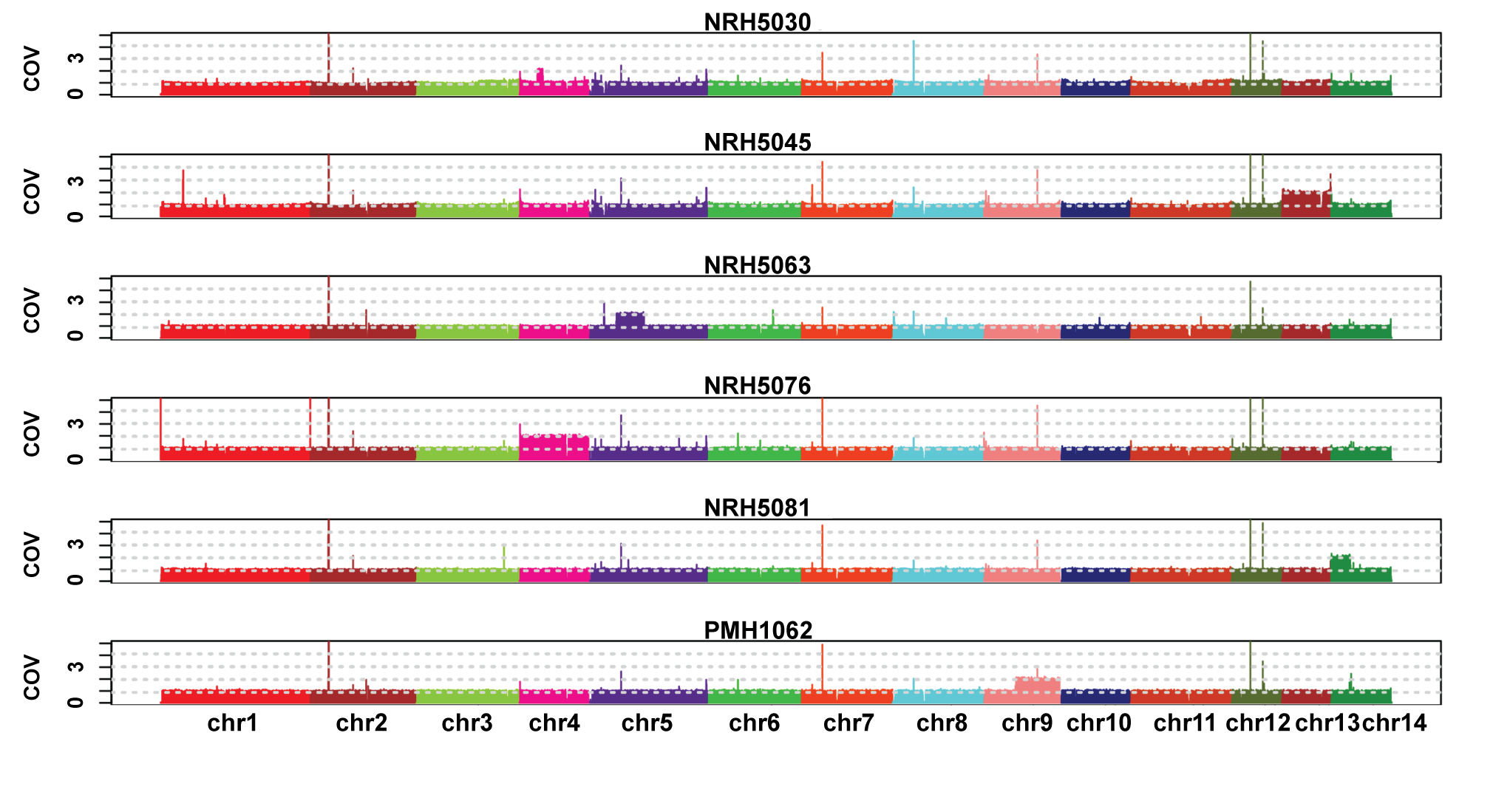

Supplement: FIG S1 [file mbio.02313-21-sf001.tif]
